# Oscillatory motor patterning is impaired in neurofibromatosis type 1: a behavioural, EEG and fMRI study

**DOI:** 10.1186/s11689-018-9230-4

**Published:** 2018-03-22

**Authors:** Gilberto Silva, Isabel Catarina Duarte, Inês Bernardino, Tânia Marques, Inês R. Violante, Miguel Castelo-Branco

**Affiliations:** 1 CNC.IBILI, Institute for Biomedical Imaging and Life Sciences, University of Coimbra, 3000-548 Coimbra, Portugal; 2 ICNAS, CIBIT, Institute for Nuclear Sciences Applied to Health, University of Coimbra, 3000-548 Coimbra, Portugal; 3 School of Psychology, Faculty of Health and Medical Sciences, University of Surrey, Guildford, GU2 7XH UK

**Keywords:** Neurofibromatosis type 1, Inhibition, EEG, fMRI, Motor coordination

## Abstract

**Background:**

Neurofibromatosis type1 (NF1) is associated with a broad range of behavioural deficits, and an imbalance between excitatory and inhibitory neurotransmission has been postulated in this disorder. Inhibition is involved in the control of frequency and stability of motor rhythms. Therefore, we aimed to explore the link between behavioural motor control, brain rhythms and brain activity, as assessed by EEG and fMRI in NF1.

**Methods:**

We studied a cohort of 21 participants with NF1 and 20 age- and gender-matched healthy controls, with a finger-tapping task requiring pacing at distinct frequencies during EEG and fMRI scans.

**Results:**

We found that task performance was significantly different between NF1 and controls, the latter showing higher tapping time precision. The time-frequency patterns at the beta sub-band (20–26 Hz) mirrored the behavioural modulations, with similar cyclic synchronization/desynchronization patterns for both groups. fMRI results showed a higher recruitment of the extrapyramidal motor system (putamen, cerebellum and red nucleus) in the control group during the fastest pacing condition.

**Conclusions:**

The present study demonstrated impaired precision in rhythmic pacing behaviour in NF1 as compared with controls. We found a decreased recruitment of the cerebellum, a structure where inhibitory interneurons are essential regulators of rhythmic synchronization, and in deep brain regions pivotally involved in motor pacing. Our findings shed light into the neural underpinnings of motor timing deficits in NF1.

## Background

Neurofibromatosis type 1 (NF1) is the most common autosomal dominant neurogenetic condition with an estimated prevalence of 1 in 3000 individuals [1–3]. The disorder is caused by mutations in the NF1 gene that encodes neurofibromin. This protein is involved in cell proliferation and differentiation [4], and its loss may explain abnormalities in the brain structure, which include increased volume of the cortical and subcortical structures, and white and gray matter abnormalities [5–9]. Behavioural difficulties are also frequent and encompass a wide range of cognitive deficits, which include perceptual impairments, attention and learning disabilities [10–12]. Moreover, there is a tendency for those symptoms to persist or even increase in severity with age [2].

Motor skill and time perception impairments have been reported in NF1 children [13]. Debrabant and colleagues [14] studied temporal perception (motor timing indexed by the reaction time decrease upon presentation of predictable stimuli). They found that the clinical group responded with an increased reaction time to such temporally predictable stimuli (as defined by regular interstimulus intervals) when compared to typically developing children [14]. Accordingly, a comprehensive study by Hyman on motor and cognitive function in NF1 showed that fine motor coordination deficits and slowing of motor speed were present in approximately 20 and 30% of the NF1 cohort, respectively [10]. Using a more broad motor performance test battery, Rietman and colleagues found that 61% of the studied cohort had motor problems [15].

Although knowledge on the cognitive and behavioural deficits in NF1 is increasing, the neural mechanisms underlying such impairments are still poorly understood. Previous studies addressing other cognitive domains have proposed that abnormalities in the balance of the excitatory and the inhibitory activity underlie a basic disease mechanism.

Initial studies in mouse models of NF1 indicated that lack of neurofibromin causes increased GABA (γ-aminobutyric acid)-mediated neurotransmission and showed a relationship between enhanced inhibitory activity and behavioural profile [16–18]. However, studies in human patients have shown a pattern of GABA alterations that include reduced GABA levels and GABA_A_ receptor density [19–21]. In order to reconcile these differences across species, a recent study investigated pre- and post-synaptic GABA levels in an NF1 mouse model employing magnetic resonance spectroscopy (the only technique available to measure GABA levels in vivo in humans) combined with molecular approaches [22]. This study showed that the pattern of GABA alterations in mice is region specific and that this pattern is not always consistent across species. Thus, although mutations in the NF1 gene do seem to impact the GABA system and those can have behavioural and cognitive consequences [21], their pattern across species and brain regions do not seem to be trivial. Importantly, lower GABA levels may still be consistent with enhanced inhibitory activity. This is the case for example in the hippocampus, where low GABA levels coexist with very high post-synaptic receptor density [22] and increased inhibitory post-synaptic potentials [18].

Here, we hypothesized that rhythmic taping performance is impaired in NF1 and that it is related to abnormal physiology and brain activity patterns. Inhibition is involved in the function of motor central pattern generators—neuronal circuits that when activated can produce rhythmic motor patterns [23]—and in the control of frequency and stability of motor rhythms [24, 25]. We therefore aimed to explore the link between behavioural control of motor rhythms, brain oscillations and brain activity in regions involved in motor pacing. To reach that goal, we choose a finger-tapping task so we can precisely measure the participant’s performance either during functional magnetic resonance imaging (fMRI) or electroencephalography (EEG). We used synchronous and asynchronous finger-tapping tasks. The latter requires alternated finger tapping, which implies interhemispheric inhibitory control, in contrast with the synchronous variant. In both synchronous and asynchronous conditions, finger tapping was performed in incremental rates, 1, 3 and 5 Hz, to vary the performance load. The latter frequency has been shown to be the most discriminative of disease states in cerebellar disorders (in particular genetic ataxias) where rhythmic motor control is impaired [26]. We hypothesized that the NF1 cohort would perform worse at rhythmic pacing than the healthy group, and we aimed to identify the neural correlates of such impaired temporal patterning. Since oscillatory pacing can be related to the modulation of populations of inhibitory interneurons [27, 28], we expected that behavioural differences would be reflected in the power of the beta band. We used the same pacing paradigm during functional scans to induce effects in the BOLD (blood-oxygen-level dependent) signal in motor-related areas during task performance. We aimed to unravel the neural underpinnings of abnormal motor coordination.

## Methods

### Subjects

Twenty-one adults with NF1 were recruited from a database used in previous studies [19] and in collaboration with the Portuguese Association of Neurofibromatosis. They all had a definite diagnosis of NF1 in accordance with the criteria of the National Institutes of Health (National Institutes of Health Consensus Development Conference, 1988). The control group (20 age- and gender-matched participants) was recruited via advertisement in the local community. Exclusion criteria for all participants included psychiatric disorders, neurologic illness affecting brain function other than NF1, brain tumor burden, intelligence quotient (IQ) lower than 75, epilepsy and traumatic brain injury. One control was excluded due to neurological illness. None of the NF1 patients were diagnosed with ADHD or had a formal diagnosis of learning disabilities. None of the participants were taking medication for treating anxiety or depression in the year before the study, and none of them were ever medicated with anticonvulsants.

All the participants but one were right-handed as assessed by the Edinburgh Handedness Inventory [29] and had normal or corrected to normal visual acuity. Intellectual function was assessed by using the Portuguese-adapted version of the Wechsler Adult Intelligence Scale-3rd edition (WAIS-III) [30]. Two control participants were unavailable to complete the IQ assessment. Full-scale IQ values were in the normal range for the NF1 patients (mean IQ ± SD 104.4 ± 13.7). All the participants performed the standard Stroop Colour and Word Test [31] composed by two congruent conditions [word (W) and colour (C)] and one incongruent (interference) condition [colour-word (CW)]. Participants had 45 s to complete each task condition. An Interference Index was calculated according to the method proposed by Golden [31]: incongruent score (IG) = CW − [(W × C)/(W + C)]. Groups did not differ regarding this index (independent-samples *t* tests, *p* > .05), indicating similar ability to control over the interference effect.

Three participants were excluded from the EEG analysis. One due to a malfunction in the trigger recording system and two due to differences in the cap system (ground positioning and channel locations). Two participants exceeded the limits of movement during the functional magnetic resonance (3 mm in, at least, one axis), and thus were excluded from that analysis. Our final sample for the EEG analysis was composed of 19 patients with NF1 and 19 controls. The clinical group (*n* = 19, 10 females, age range 23.8–51.8, mean age ± standard deviation [SD] 36.1 ± 6.7) and the control group (*n* = 19, 11 females, age range 22.5–55.0, mean age ± SD 37.1 ± 7.2) were matched for age (*U* = 160.0, *p* = 0.549) and gender (*χ*^2^ (1) = .106, *p* = 0.744). The final sample for the fMRI analysis was composed of 19 patients with NF1 and 20 controls. The clinical group (*n* = 19, 11 females, age range 23.8–51.8, mean age ± standard deviation [SD] 36.5 ± 7.0) and the control group (*n* = 20, 12 females, age range 22.5–55.0, mean age ± SD 36.8 ± 7.1) were matched for age (*U* = 182.5, *p* = 0.833) and gender (*χ*^2^ (1) = .018, *p* = 0.894).

### Task

Participants performed a previously validated audio-paced tapping paradigm [26] at three incremental frequencies (1, 3 and 5 Hz). Participants were asked to tap using both index fingers either simultaneously (“synchronous” condition) or alternating the tapping (“alternating” or “asynchronous” condition) as prompted by a cue shown in a computer screen (“A” for the alternating condition and an “S” for the synchronous condition). All participants were familiarized with the task and the interface before the beginning of the recording sessions. The paradigm was designed and presented using the Psychophysics Toolbox 3 [32, 33], running on Matlab R2013b (MathWorks, Natick, MA, USA).

For the fMRI task, a total of 24 blocks of the motor paradigm, 8 per frequency (4 synchronous and 4 alternating), were presented. Block duration was 9 s either for the finger tapping or for the baseline. The cues were presented in a 698.40 × 392.85 mm LCD monitor (NordicNeuroLab, Bergen, Norway), placed ~ 156 cm away from the participants’ head. Audio was provided through MR-compatible headphones. Behavioural data (tapping timings) were recorded using the MRI-compatible response box Lumina LP-400 (Cedrus Corporation, San Pedro, CA, USA).

**Fig. 1:**
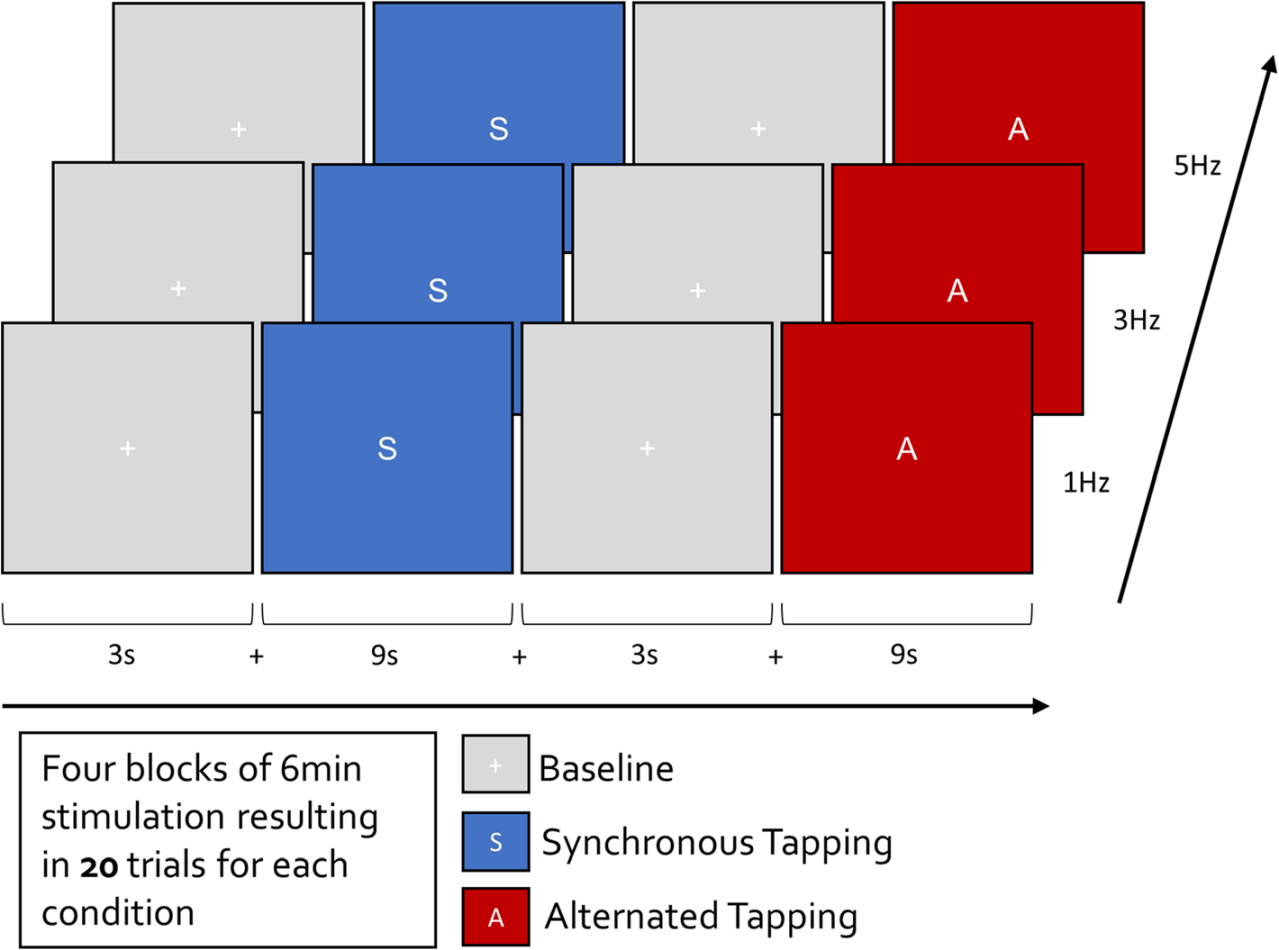
Experimental design of the behavioural task during the EEG task (concerning the fMRI design, see text). The participants were instructed to make an audio-paced tapping using both index fingers, synchronously or alternately, as indicated by the visual cue. The tapping frequency was set at 1, 3 or 5 Hz, and it was set by a beep

Regarding the EEG acquisition, we acquired four runs, each composed of five repetitions of the main sequence (1, 3 and 5 Hz, each frequency executed in two variations, synchronous and alternating). Blocks lasted 12 s and were composed by a baseline period of 3 s (where the participant fixated the central cross and did not execute any movements) and 9 s of task (see Fig. 1). The cueing paradigm was presented in a laptop placed ~ 50 cm in front of the participant. Participants were instructed to fixate the centre of the screen during the EEG task, and behavioural data were recorded using the “Z” and “M” buttons of a common keyboard.

### MR recordings and analysis

MR scans were acquired in a 3T Magnetom Tim Trio scanner (Siemens, Erlangen, Germany), using a 12-channel birdcage head coil. A T1-weighted magnetization-prepared rapid gradient echo sequence was acquired with a repetition time of 2530 ms, echo time of 3.42 ms, resolution of 1 mm^3^ isotropic voxel, flip angle of 7°, matrix size 256 × 256 and a field of view of 256 × 256 mm. Functional data were acquired using echo-planar imaging sequences, using voxel size 3 mm^2^ and slice thickness of 3 mm, no gap between slices, 43 slices acquired parallel to the anterior commissure-posterior commissure line, repetition time 3000 ms, echo time 30 ms, flip angle of 90°, matrix size 256 × 256 and a field of view of 256 × 256 mm. In total, 147 volumes were acquired. A T2-weighted fluid attenuation inversion recovery sequence was used to identify unidentified bright objects, and it was acquired using a 1 mm^3^ voxel, repetition time 5 s, echo time 388 ms, inversion time 1.8 s, field of view 250 × 250 mm, matrix size 256 × 256 and160 slices.

The complete processing pipeline and analysis of the functional data was done using BrainVoyager QX 2.8.2 (Brain Innovation, Maastricht, The Netherlands). Data were corrected for (1) slice scanning time differences using cubic spline interpolation, (2) motion artifacts by combining trilinear and sinc function based methods for interpolation in the three axes and (3) filtered in the time domain using an approach with Fourier basis set using 2 cycles per time course. Functional data were automatically co-registered to the anatomical T1 (and manually verified) and subsequently normalized to the Talairach atlas. Spatial smoothing was applied using a Gaussian kernel with a full width at half maximum of 6 mm. Statistical analyses were performed at the group level using a general linear model approach. The predictor’s model was obtained by convolution of the boxcar function with a standard 2-gamma hemodynamic response function. Motion parameters were also included in the model as regressors of no interest. Random effects analysis (RFX) was performed, and the results were corrected for multiple comparisons using false discovery rate (FDR) with a fixed *p* value of 0.05 and minimum cluster extension of 20 voxels.

### EEG recordings and analysis

Electroencephalographic signals were recorded using a 64 electrodes cap (QuickCap, NeuroScan, USA) with electrodes placed according to the extended 10/20 system. In order to ensure the quality of the signal, all electrode impedances were kept below 20 kΩ. The continuous signal was amplified and recorded at a sampling rate of 1 kHz, low pass filter at 200 Hz, through a SynAmps2/RT amplifier. Data were acquired using Scan 4.5 (NeuroScan, Compumedics, Charlotte, NC), and the acquisition reference was set to an electrode located at a half distance between CZ and FCZ.

The processing was performed using the EEGlab toolbox [34] for Matlab (MathWorks, Natick, MA). The EEG signal was downsampled to 400 Hz and digitally filtered between 1 and 100 Hz. A notch filter (47.5–52.5 Hz) was applied, and epochs from − 3000 to 9000 ms were obtained locked to the stimuli onset. Epoch rejection was done automatically by scanning the entire dataset using a rejection threshold of 120 μV for all electrodes followed by visual inspection to ensure the data was free from artifacts. Channels with abnormal noise activity were interpolated using spherical spline interpolation. The HEO/VEO channels were excluded from further analysis, and the recordings were re-referenced to the average of all remaining channels. We proceeded to epoch division by trigger data, i.e. we separated the time-frequency epochs by sub-task type (1, 3 and 5 Hz tapping, synchronous and alternated). All the participants met the minimum criterion (more than 40%) of the trials per condition available for analysis.

Time-frequency decomposition was done using the function *pop_newtimef()*, with Morlet wavelet, beginning with a 7-cycle at 6 Hz and increasing linearly with frequency (maximum of 30 cycles at 50 Hz). We calculated 400 timepoints (with an effective time window from − 2600 to 6080 ms) and set the baseline from the beginning of the epoch until 100 ms before the onset of the stimulus. Electrodes corresponding to motor areas were clustered (FC1, FC2, CZ, C1, C3, C2 and C4). We truncated the time analysis intervals (from − 2000 to 6000 ms) in order to avoid boundary effects on time-frequency spectra.

Further analysis was performed in the time-course variation for three sub-bands of mu, beta and gamma frequencies (respectively, 8–12, 20–26 and 40–44 Hz intervals). Beta range (20–26 Hz range) was defined around the desynchronization peak that matches the motor pattern. The choice of the interval of low gamma was based on our previous work [35–37] and was set up to 44 Hz to avoid power line interferences in the time-frequency analysis. The bands were not juxtaposed, in order to avoid information contamination between bands. Frequency-domain evaluations were performed specifically for beta sub-band variations, since they exhibit a marked sinusoidal profile, matching motor responses. For that, we computed the power per subject at the exact frequency of tapping.

### Statistics for group comparisons

Measurements of motor performance were done in data recorded during EEG recordings because larger amounts of data could be recorded. The statistical comparison between groups was performed using one-way multivariate ANOVAs (MANOVAs) to determine whether there are any differences between the independent variable (group) based on the dependent continuous variables (tapping at three different frequencies: 1, 3 and 5 Hz), for synchronous and asynchronous conditions. Similarly, we used MANOVA to test group differences on EEG data.

## Results

### Behavioural results

**Fig. 2:**
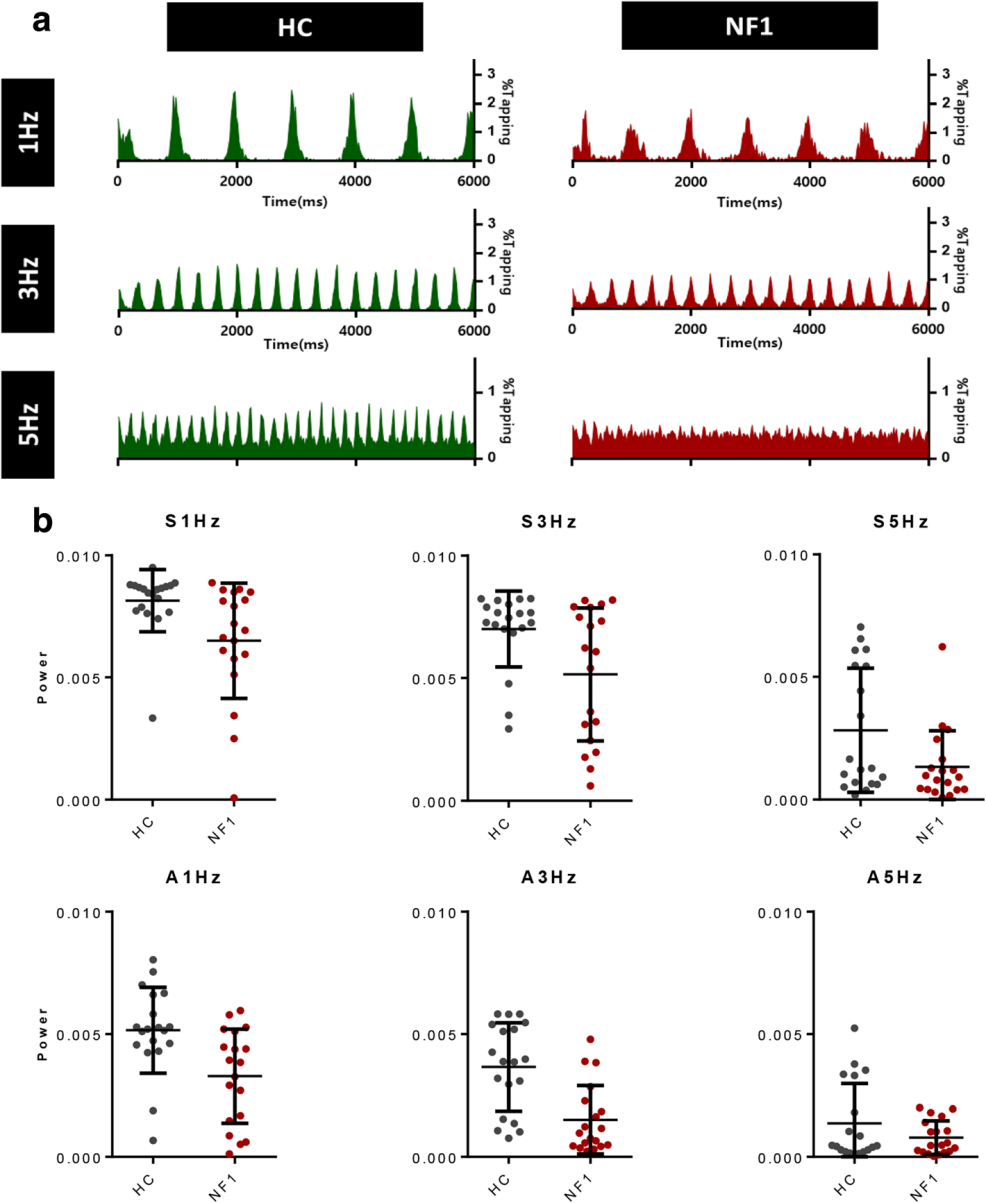
**a** Tapping time histograms (relative tapping frequency) for the synchronous condition. **b** Power at the ideal (cues) tapping frequency for controls and NF1 for the synchronous (S) and alternating (A) conditions. Healthy controls (green) performed better than participants with NF1 (red) at all the conditions, except the 5 Hz condition. The horizontal lines indicate the mean and standard deviation

Tapping time histograms reflecting the distribution of motor responses in the synchronous condition are presented in Fig. 2. The histograms indicate a reduction in tapping time precision in patients with NF1 compared to the healthy control group as indexed by the sharper curve around the cued tapping time in the control group. To quantify this effect, we computed the power at the ideal tapping frequency as a measure of motor performance precision. Higher power values correspond to higher participant’s ability to keep tapping at the required frequency.

For the synchronous condition, we found, as assessed by MANOVA, a statistically significant difference between controls and NF1 patients, *F* (3, 34) = 6.10, *p* = 0.041; Wilk’s *Λ* = 0.787. These differences in behavioural performance were significant at all tested frequencies of 1, 3 and 5 Hz (respectively, *F* (1, 36) = 7.14, *p* = 0.011; *F* (1, 36) = 6.61, *p* = 0.014; and *F* (1, 36) = 4.887, *p* < 0.034).

For the asynchronous condition, MANOVA also yielded statistically significant differences between groups, *F* (3,34) = 6.104, *p* = 0.02; Wilk’s *Λ* = 0.650. Subsequent analyses revealed that these effects were mainly derived from differences at 1 and 3 Hz (respectively, *F* (1, 36) = 9.84, *p* = 0.003 and *F* (1, 36) = 17.01, *p* < 0.001).

**Fig. 3:**
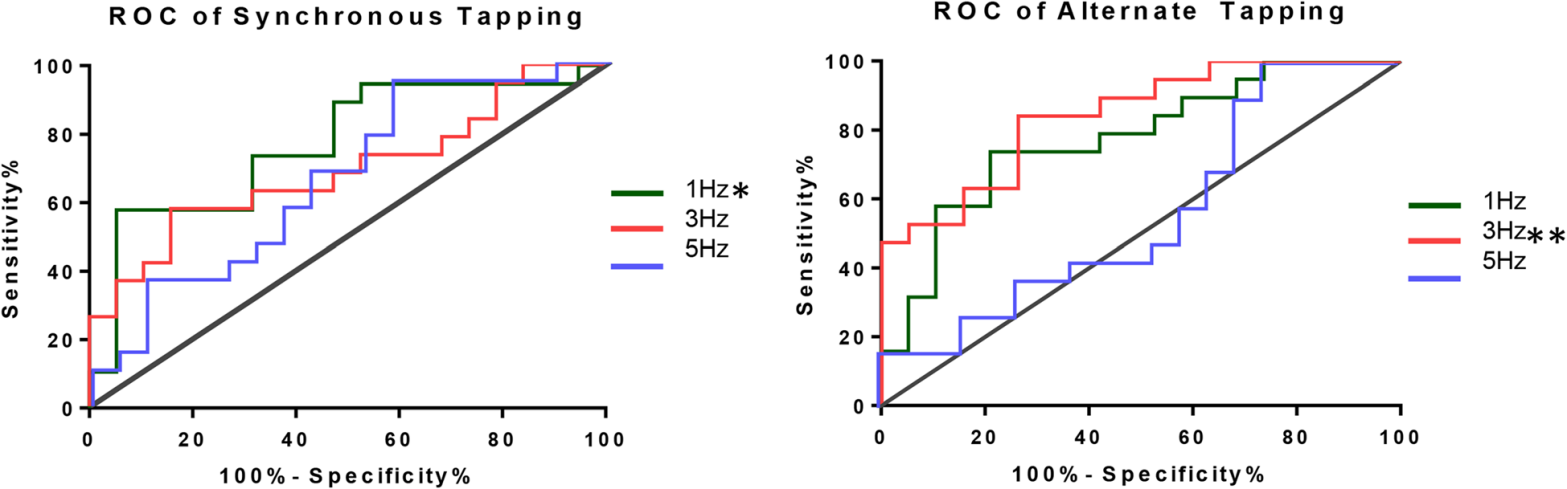
Sensitivity and specificity analysis of the power at the expected frequency of tapping. ROC curves were computed for both synchronous and alternating conditions at every frequency of finger tapping (1, 3 and 5 Hz). The best results were found for alternated tapping at 3 Hz (**), which ROC curve showed a sensitivity of 84% and a specificity of 74%, and for the synchronous tapping at 1 Hz (*) showing a sensitivity of 74% and a specificity of 47% to discriminate patients with NF1 from healthy controls

We followed up this analysis by performing ROC (receiver operating characteristic) curve analysis to further investigate which frequencies better discriminated between patients (*n* = 18) and controls (*n* = 19), Fig. 3. Synchronous 1 Hz tapping and alternated 3 Hz tapping were the most suitable to separate patients with NF1 from controls (respectively, area ± standard deviation 0.7729 ± 0.0781, *p* = 0.012 and 0.8421 ± 0.0622, *p* < 0.01, corrected for multiple comparisons), such that the alternated test at 3 Hz achieved a sensitivity of 84% with 74% specificity.

### EEG results

**Fig. 4:**
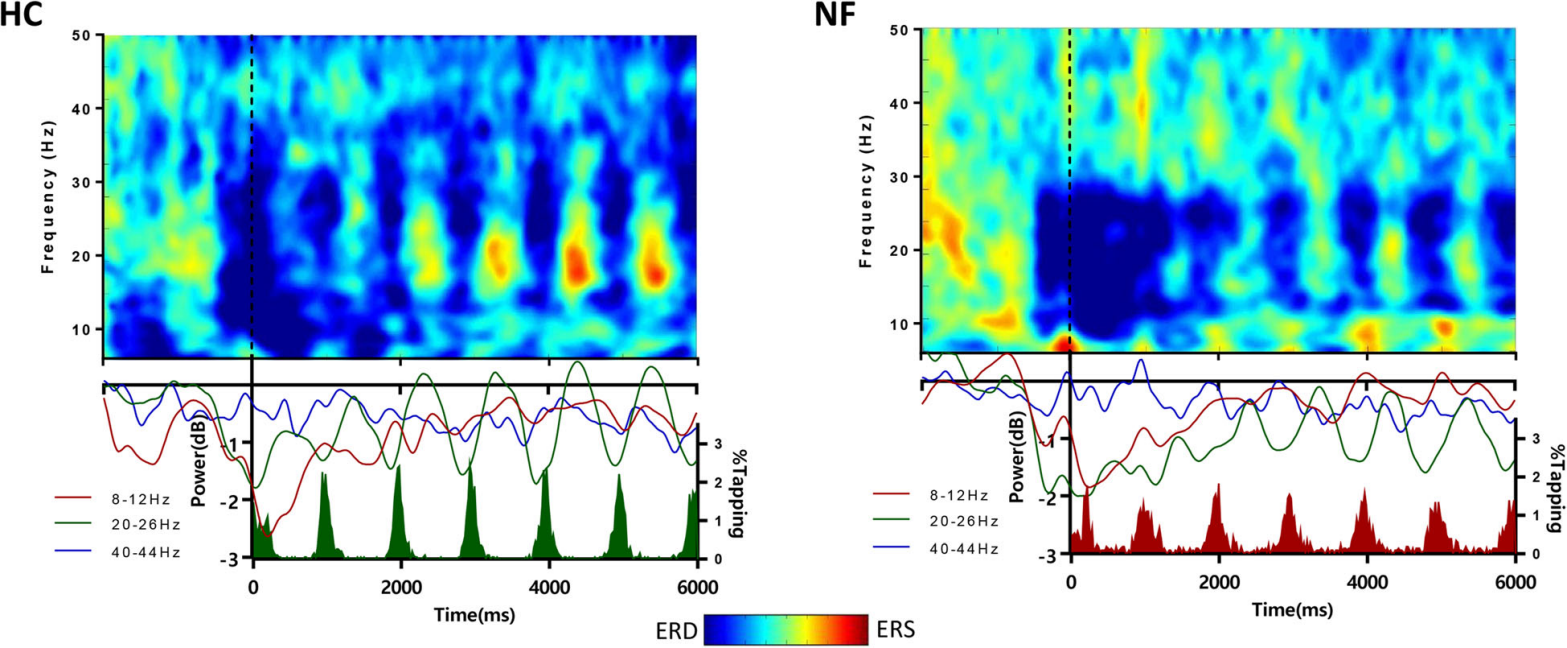
Time-frequency plots of the control and NF1 groups during synchronous finger tapping at 1 Hz. A similarly strong periodical variation in the beta band is conspicuous in both groups and at the ideal motor tapping frequency in the beta sub-band of 20–26 Hz, centred in the desynchronization beta peak (23 ± 3 Hz) and with a modulation matching behaviour. Note that the colour peaks just reflect maxima and minima positions, and it is the difference that needs to be considered for statistical analysis

Time-frequency analysis in the motor cluster (electrodes FC1, FC2, CZ, C1, C3, C2 and C4) was performed for both groups. Plots for the synchronous condition at 1 Hz are presented in Fig. 4. Both groups exhibit a marked cyclic synchronization/desynchronization pattern in the *mu* and *beta* range, which are known to be tightly related with the behavioural execution of movements.

To statistically compare the groups concerning this synchronization/desynchronization pattern in the 8–12 Hz (corresponding to mu band) and 20–26 Hz range (the peak of beta range, corresponding to the desynchronization peak matching the motor pattern, see above), we computed the power for the specific frequency of the task per subject. We did not find statistical differences in the power between groups across any of these motor pacing frequencies.

### fMRI results

**Fig. 5:**
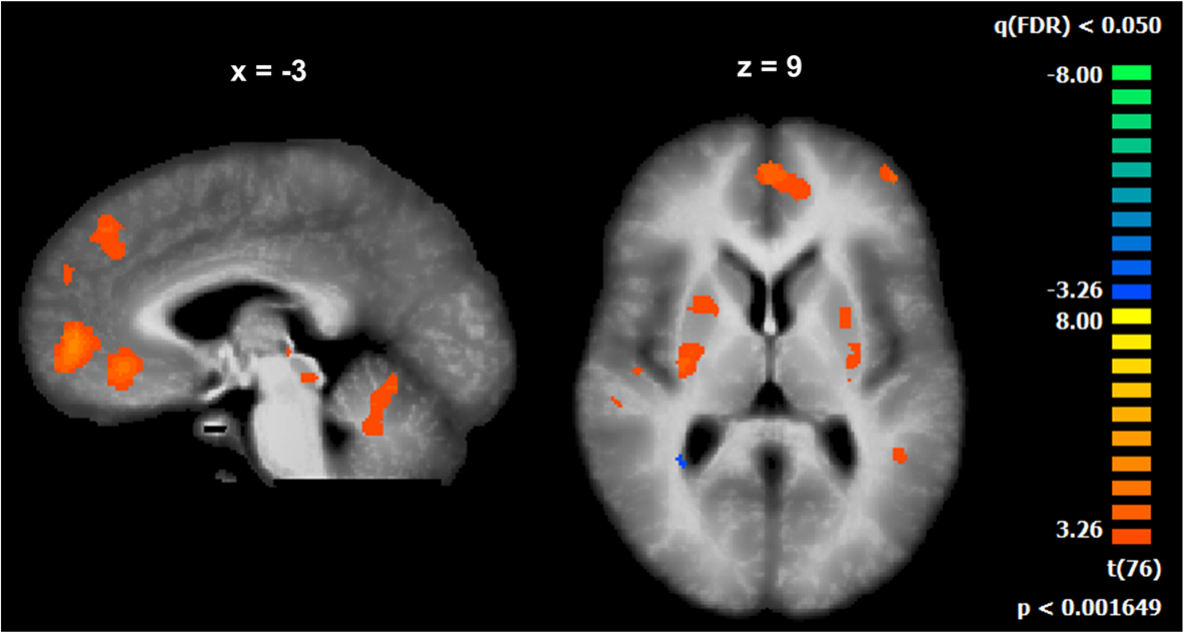
Significant differences between the control group and the NF1 group (*t*(76) > 3.26, *p* < 0.05, FDR corrected for multiple comparisons, minimum cluster size of 20 voxels) during performance matched conditions (5 Hz synchronous and alternate)

Our analysis only identified differences between healthy controls and patients with NF1 at 5 Hz, as presented in Fig. 5 (*t*(76) > 3.26, *p* < 0.05, multiple comparison FDR corrected, minimum cluster size of 20 voxels). The control group presented a higher recruitment of the putamen, cerebellum (anterior lobe), red nucleus, medial prefrontal cortex and auditory cortex, bilaterally. The left superior parietal lobule showed higher activation in the group of patients with NF1 than controls. All findings are detailed in Table 1.

**Table 1:** Regions differentially recruited by the control group and the NF1 group (*t*(76) > 3.26, *p* < 0.05, FDR corrected for multiple comparisons, minimum cluster size of 20 voxels) during the fastest tapping conditions (5 Hz synchronous and 5 Hz alternate tapping). The clusters are described by their hemisphere (H), peak voxel coordinates in Talairach space, the *t* and *p* values in the peak voxel and the number of voxels (*n*)

		Peak					
Region	H	*x*	*y*	*z*	*t*	*p*	*n*
Putamen	R	30	− 13	7	4.30	0.000027	33
Putamen	L	− 30	− 10	14	3.93	0.000119	25
Cerebellum	R	15	− 40	− 26	4.02	0.000084	39
Cerebellum	R,L	0	− 52	− 26	3.90	0.000135	40
Red nucleus	R,L	9	− 19	− 2	4.75	0.000004	55
Medial prefrontal cortex	R, L	− 3	50	4	5.16	0.000001	223
Medial frontal gyrus	L	− 9	29	31	4.24	0.000035	92
Middle frontal gyrus	L	− 24	17	43	4.58	0.000008	28
Middle frontal gyrus	L	− 43	50	7	4.35	0.000022	47
Posterior cingulate gyrus	L	− 9	− 49	32	3.71	0.000275	26
Auditory cortex	R	51	− 28	16	5.16	0.000001	75
Auditory cortex	L	− 42	− 28	19	5.80	< 0.000001	57
Superior temporal gyrus	R	57	− 10	1	5.34	< 0.000001	36
Superior temporal gyrus	L	− 57	− 43	− 8	4.44	0.000015	92
Middle temporal gyrus	L	− 60	− 22	− 20	5.43	< 0.000001	217
Parahippocampal gyrus	L	− 27	5	− 26	4.46	0.000014	54
Precentral gyrus	R	15	− 28	67	5.88	< 0.000001	26
Superior parietal lobule	L	− 30	− 58	43	− 4.57	0.000009	50
White matter	R	24	− 46	15	− 4.62	0.000007	39
White matter	L	− 27	20	25	4.17	0.000047	33

## Discussion

In the present study, we aimed to understand the neural basis of rhythmic motor pacing deficits in NF1, using a comprehensive set of approaches, including behavioural assessment, EEG and fMRI. We studied a cohort of participants with NF1 and healthy controls during a simple motor task requiring pacing at distinct frequencies during EEG recording and fMRI scans.

We found that NF1 patients were significantly impaired in the behavioural precision of rhythmic pacing. Their tapping times showed larger dispersion and therefore decreased power at the cued pacing frequency. Time-frequency analysis revealed similar oscillatory patterns across groups that mirrored motor behaviour. Accordingly, the power at the beta sub-band matched the motor behavioural patterning. BOLD signals evoked by the task suggested group differences in the deep brain regions pivotally involved in motor pacing, not reachable by EEG, such as the basal ganglia and the cerebellum.

### Power at the cued frequency as potentially relevant clinical measure

The histograms of tapping times of both groups showed clear group differences. The tapping distribution of the healthy control group resulted in sharper curves with higher amplitude near the ideal (cued) tapping frequency. As the responses were sparser in time, the curve of NF1 participants was broader and, consequently, the amplitude was lower than the control group curve. The statistical analysis of the power at the cued tapping frequency demonstrated that performance was statistically different between groups across all frequencies in the synchronous task and at 1 and 3 Hz in the asynchronous task. ROC curve analysis showed the best discrimination for alternate tapping at 3 Hz. This analysis showed a sensitivity of 84% and a specificity of 74% to discriminate patients with NF1 from healthy controls. The alternate tapping variant poses larger cognitive control demands and inhibition from the contralateral hemisphere as opposed to the synchronous tapping. Our results suggest that the combination of 3 Hz and the alternate variant task rendered this task more sensitive to detect impairments in the NF1 cohort.

Impaired performance during motor tests has been often reported in NF1 [10, 13–15, 38–41] but not in the context of rhythmic pacing. Along with the motor deficits, a wide array of impairments in other cognitive domains is known, as executive dysfunction [3, 10, 39], and it is believed that there is a common biological mechanism underlying all these deficits. There is however the concern whether executive dysfunction could underlie observed motor deficits [40, 41]. However, this is an unlikely explanation for our results because our groups were matched in executive function, as measured by a Stroop task. Moreover, we used a task without working memory load and no need to memorize motor sequences.

Studies on mouse models suggest that abnormal neurodevelopmental mechanisms in NF1 can result from an imbalance on the excitatory and the inhibitory drive [16, 18, 22], and this may also be the case for the observed changes in rhythmic motor pacing.

### Cyclic pattern of behavioural and EEG power curves

The time-frequency analysis of the signal over the sensorimotor cortex showed a strong periodical variation in the beta band, which closely matches the behavioural data. This is a well-established spectral pattern (including also the so-called post-movement beta rebound); beta oscillations over the motor cortex showed increased desynchronization at the beginning of a movement and increased synchronization after the movement (~ 300 to 1000 ms) [42]. Therefore, a rhythmic finger-tapping task was expected to produce a synchronization/desynchronization cyclic pattern in the beta band with the same frequency as the tapping movement.

A relation between beta oscillations and inhibitory activity was demonstrated before by Gaetz and colleagues. They found that the level of GABA, an inhibitory neurotransmitter, in the motor cortex correlated with the power of the post-movement beta transient increase [27]. Jensen and co-workers further found that benzodiazepines, which are GABAergic agonists, modulate beta sources by increasing the power of the beta oscillations at rest over the primary sensorimotor cortex [28]. Modulations in beta oscillations are linked to the behaviour of inhibitory interneurons and thus have been related to the underlying excitation/inhibition balance [42, 43]. Future work should further test whether this putative link between GABA and abnormal beta activity is suggestive of impaired cortical inhibition and can be related to the behavioural impairments we found here.

### A critical role for regions involved in motor pacing: the basal ganglia and cerebellum

Functional neuroimaging data analysis showed group differences at the fastest frequency (5 Hz). This pacing rhythm was previously demonstrated to best discriminate between healthy participants and patients with genetic disorders leading to cerebellar atrophy and impaired rhythmic motor control [26]. Here, the group contrast revealed higher recruitment of subcortical structures of the extrapyramidal motor system in the control group, namely the putamen, cerebellum and red nucleus. The synchronization between the audio pacing stimuli and the motor action requires complex information processing involving diverse functions such as timing, temporal prediction, sequence processing and sensory-motor integration [44]. This implies the recruitment of a wide network involving the primary auditory cortex, motor cortex, basal ganglia and cerebellar circuits. The putamen, as part of basal ganglia, plays an important role in motor control [45], and it is known to be parametrically modulated by movement frequency [46]. The red nucleus is a pivotal region in motor function and shows significant functional connectivity with the cerebellum [47]. It is also generally accepted that the cerebellum plays a role in rhythmic synchronization, by processing timing information, as required by the present task [44, 48]. In the cerebellum, inhibitory interneurons are essential regulators of motor coordination [49]. The pattern of differences found in the cerebellum, a structure which is dominated by inhibitory physiology, is consistent with the inhibitory/excitatory imbalance theory in NF1. The task required the participant to keep continuous monitoring of his/her own motor pacing performance, which may explain the activation of the dorsolateral prefrontal regions. As the level of motor demand increases, the effort required to perform the task also increases. It is known that there is an increase in prefrontal cortex activity with the increasing of the cognitive control demands [50] that is intrinsically related to the role of PFC in monitoring and top-down control [51]. This may explain the observed differential activation in fronto-striatal networks in our study.

The group differences identified for the auditory cortex and superior parietal lobe are quite intriguing. Sensorimotor synchronization implies pacing and time prediction. As our motor paradigm is audio paced, this process starts with the sensory input and with attentional deployment in parietal cortex. This suggests that sensorimotor integration and synchronization are also impaired in NF1.

## Conclusions

In the present study, we aimed to investigate the neural basis of putative rhythmic motor pacing deficits in NF1 by comprehensively exploring behavioural motor control, brain rhythms and brain activity in neurofibromatosis type 1.

Our study demonstrates impaired precision in rhythmic pacing behaviour and sheds light into the neural underpinnings of motor timing deficits in NF1, in particular concerning the basal ganglia and the cerebellum.

## Abbreviations

BOLD: Blood-oxygen-level dependent
EEG: Electroencephalography
FDR: False discovery rate
fMRI: Functional magnetic resonance imaging
GABA: γ-Aminobutyric acid
IQ: Intelligence quotient
NF1: Neurofibromatosis type 1
RFX: Random effects analysis
ROC: Receiver operating characteristic
SD: Standard deviation
